# Guided Bone Regeneration Using a Modified Occlusive Barrier with a Window: A Case Report

**DOI:** 10.3390/biomimetics10060386

**Published:** 2025-06-10

**Authors:** Luis Leiva-Gea, Alfonso Lendínez-Jurado, Paulino Sánchez-Palomino, Bendición Delgado-Ramos, María Daniela Corte-Torres, Isabel Leiva-Gea, Antonio Leiva-Gea

**Affiliations:** 1 Clínicas ClearDent, 23006 Jaén, Spain; leiva1987@hotmail.com; 2 Facultad de Odontología, Universidad de Murcia, 30003 Murcia, Spain; 3 Hospital Regional Universitario de Málaga, 29011 Málaga, Spain; 4 Facultad de Medicina, Universidad de Málaga, Andalucía Tech, Campus de Teatinos s/n, 29071 Málaga, Spain; 5 Instituto de Investigación Biomédica de Málaga (IBIMA)-Plataforma BIONAND, 29010 Málaga, Spain; antonioleivagea7@gmail.com; 6 Facultad de Odontología, Universidad de Granada, 18011 Granada, Spain; paulinosanchezp@gmail.com (P.S.-P.);; 7 Biobanco del Principado de Asturias, FINBA-ISPA, 33011 Oviedo, Spain; mdanielac@hotmail.com; 8 Departamento de Biomedicina y Odontología, Facultad de Ciencias Biomédicas y Deporte, Universidad Europea de Andalucía, 29010 Málaga, Spain; 9 Hospital Universitario Virgen de la Victoria, 29010 Málaga, Spain

**Keywords:** guided bone regeneration, titanium barriers, bone augmentation, dental implants

## Abstract

Background: Bone resorption following tooth loss poses significant challenges for dental implant success. Guided bone regeneration (GBR) techniques, particularly in vertically deficient ridges, often require complex procedures and soft tissue management. This case report introduces a modified occlusive barrier with a window, combined with tricalcium phosphate, to address these challenges. Methods: A 26-year-old female with significant bone loss in the mandibular anterior region underwent GBR using a digitally designed titanium occlusive barrier. The barrier was fabricated using CAD/CAM technology and secured with screws. A blood clot mixed with tricalcium phosphate was used to promote bone regeneration. Postoperative care included regular irrigation, de-epithelialization, and follow-up over six months. Implant placement and histological analysis were performed to evaluate outcomes. Case Presentation: The patient achieved 8.8 mm of vertical and 7.6 mm of horizontal bone regeneration. Histological analysis confirmed the presence of mature, mineralized bone, and keratinized gingiva. The implant was successfully placed, and a fixed prosthesis was restored after four months, with stable results at a three-year follow-up. Conclusion: This technique demonstrates effective bone and soft tissue regeneration in a single procedure, eliminating the need for autologous bone grafts and secondary surgeries. The use of a digitally designed occlusive barrier offers precision, reduces morbidity, and simplifies the surgical process, suggesting a promising advancement in GBR. Further studies are needed to validate these findings.

## 1. Introduction

Bone resorption following tooth loss presents significant challenges for dental implant success, with both horizontal and vertical bone reduction occurring within initial months and potentially progressing for years [1].

The concept of guided bone regeneration (GBR) traces its origins to the pioneering work of Nyman et al. [2], who first demonstrated the potential of barrier membranes to selectively guide tissue formation, a principle later adapted for alveolar ridge preservation [3]. Contemporary techniques for bone regeneration [4] face particular challenges in vertical augmentation cases [5], often requiring supplemental soft tissue grafts [6,7,8] to ensure optimal implant outcomes. The integration of digital workflows has revolutionized GBR, improving surgical precision, and predictability while minimizing complications [9,10].

This approach aligns with recent advances in the development of artificial periostea, where biomimetic designs integrating biochemical and biophysical cues have shown significant potential to enhance bone healing and neovascularization. Moreover, the use of customized, 3D-printed titanium meshes in the context of GBR exemplifies the integration of digital workflows to create patient-specific structures capable of maintaining space and supporting vascularized bone formation. These innovations reflect the interdisciplinary nature of bionics, combining principles from biology, engineering, and materials science to develop advanced therapeutic solutions [11].

Building upon these historical foundations, we present a novel approach using a customized titanium occlusive barrier (OSTEOPHOENIX^®^, Osteophoenix SL., Vizcaya, Spain) [12,13] with structural modifications, combined with a tricalcium phosphate-enhanced blood clot matrix—a biomaterial combination whose regenerative potential has been recently documented [14].

While existing literature on such occlusive barrier systems remains limited [12,13,14,15], this case report aims to demonstrate their clinical applicability, particularly in addressing the historical limitations of conventional GBR techniques in complex ridge augmentation scenarios.

## 2. Materials and Methods

### 2.1. Case Report

A 26-year-old woman presented with significant bone loss in both height and width in the region of teeth 31 and 41. She was evaluated for regenerative treatment and implant placement in the affected area at a private practice (ClearDent) in Jaén, Spain.

### 2.2. Ethics

The study protocol adhered to the principles outlined in the Declaration of Helsinki and received approval from the Research Ethics Committee of Jaén (Ref. 1794-N-22). Written informed consent was obtained from all participating patients, encompassing both the surgical intervention and their inclusion in the study. All data were anonymized to ensure confidentiality.

### 2.3. Preoperative Information

**Figure 1:**
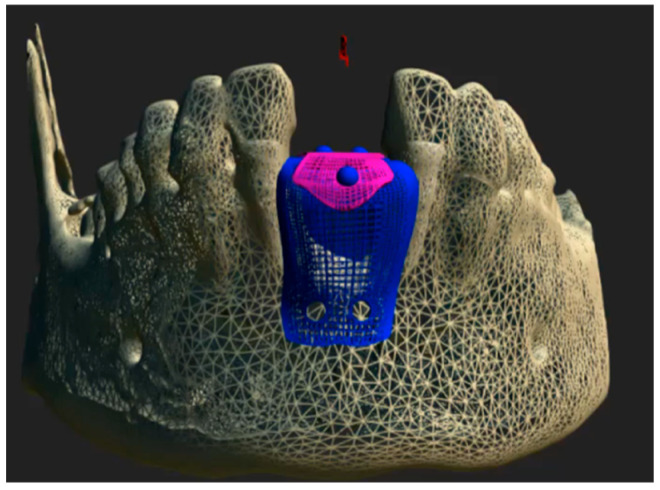
Computer-aided design (CAD) of the customized Ti6Al4V occlusive barrier.

After the medical history, oral photographs taken with a camera, intraoral scanning (PRIMESCAN^®^, Dentsply Sirona Iberia, Barcelona, Spain), and low-dose cone beam computed tomography (CBCT), as well as the evaluation of the dental treatment, an occlusive barrier was fabricated to regenerate the affected area (Figure 1).

CBCT images were acquired in Digital Imaging and Communications in Medicine (DICOM^®^) format and subsequently processed using ScanIP^®^ (Synopsys, Sunnyvale, CA, USA) software (version 7.0). A three-dimensional mask was generated and exported in stereolithography (.STL) file format. The resulting STL file was then imported into Rhinoceros^®^ 3D (Asuni Soft S.L., Barcelona, Spain) modeling software (version 6.0), where the Osteophoenix^®^ titanium barrier was digitally designed in three dimensions. The Ti6Al4V barrier was fabricated via laser sintering at approximately 350 °C (660 °F, porosity 30–60 µm) with final dimensions of 12.20 mm (width) × 14.17 mm (length) × 21.28 mm (height) and 0.7 mm thickness. Subsequently, three perforations were made: two on the buccal side and one on the lingual side, each with a diameter of 2.1 mm. This was followed by pink anodization, and then milling using CAM Magics 23 software, with a final sintering process at 1450 °C. Finally, the barrier was sterilized in an Euronda E10 autoclave at 134 °C.

## 3. Surgical Protocol

### 3.1. First Surgical Phase

#### 3.1.1. Barrier Placement

Prior to the surgical procedure, venous blood was collected from the patient for subsequent clot formation. Local infiltrative anesthesia was administered using 4% articaine in the operative field. A carefully planned incision was made with a 15c scalpel, extending from tooth 32 to 42 in the anterior mandibular region, incorporating the mesial papillae of both teeth and including apical releasing incisions. Following full-thickness flap elevation to expose the alveolar bone, cortical perforations were performed to enhance angiogenesis [16].

The surgical site was prepared using a standardized drilling protocol: initial perforation with a 1.0 mm diameter drill at 450 rpm under sterile saline irrigation, followed by final preparation with a 1.3 mm diameter drill. The titanium occlusive barrier was then secured using three fixation screws (1.5 mm diameter × 9 mm length). The autologous blood clot, previously fragmented and mixed with tricalcium phosphate [15], was carefully transferred to the regeneration site.

**Figure 2:**
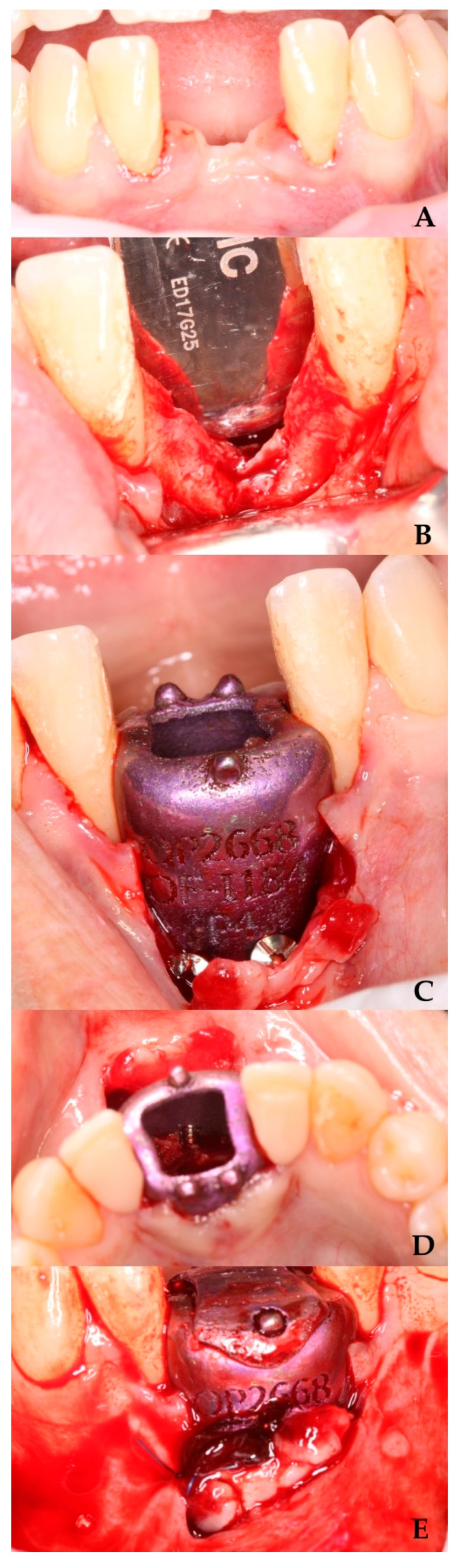
Preparation of the area for GBR with the barrier. (**A**) Area for barrier placement. (**B**) Full-thickness flap elevation. (**C**) Barrier fixation with screws. (**D**) Area for introducing the clot with tricalcium phosphate. (**E**) Suture of the releasing incision.

**Figure 3:**
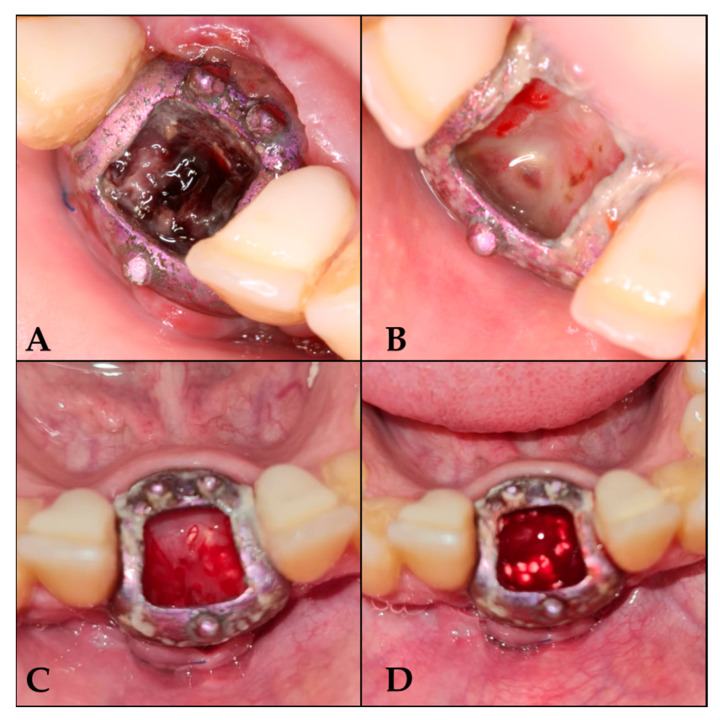
Follow-up sequence. (**A**) 7 days after surgery; (**B**) 16 days after surgery; (**C**) 25 days after surgery; (**D**) 42 days after surgery, once de-epithelialized and with tricalcium phosphate.

Closure was achieved through a combination of interrupted 3-0 non-absorbable sutures and approximation sutures at the crestal incision. The releasing incisions were subsequently sutured (Figure 2 and Figure 3A), completing the surgical procedure.

#### 3.1.2. Postoperative Care

After surgery, antibiotic therapy was initiated with amoxicillin–clavulanic acid, along with prescribed analgesics for one week.

- Day 7: Irrigation with saline solution and removal of loose debris.
- Day 16: Loss of osteoid volume and necrotic blood was observed. De-epithelialization was performed without anesthesia to induce bleeding, and tricalcium phosphate was reapplied once the blood had coagulated.
- Days 25, 33, 42, 50, and 59: Repetition of the irrigation and de-epithelialization protocol.
- From the third month onwards: Follow-up visits every 21 days until 6 months, with irrigation protocol only (Figure 3).

#### 3.1.3. Barrier Removal Day

**Figure 4:**
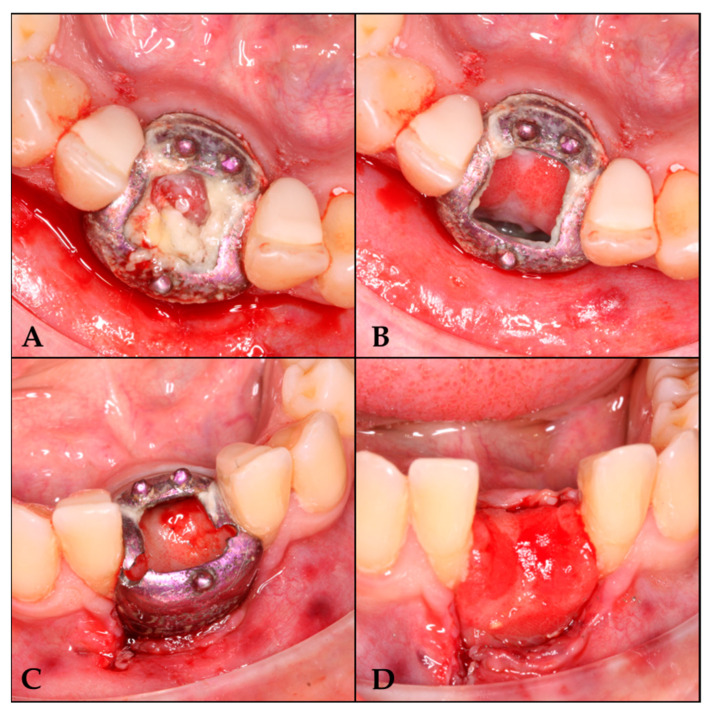
Barrier removal. (**A**) Lifting of the cover. (**B**) Area irrigated with physiological saline solution. (**C**) Barrier section. (**D**) Regenerated area.

A six-month follow-up period was conducted prior to the placement of the barrier. Anesthesia was administered, the screws were removed, and the barrier was sectioned into two segments for extraction (Figure 4).

### 3.2. Second Surgical Phase

Following a four-month healing period, implant placement was initiated under local anesthesia. A computer-guided surgical stent was utilized to achieve optimal prosthetic positioning. A bone core sample was then harvested using a trephine drill for histological analysis.

**Figure 5:**
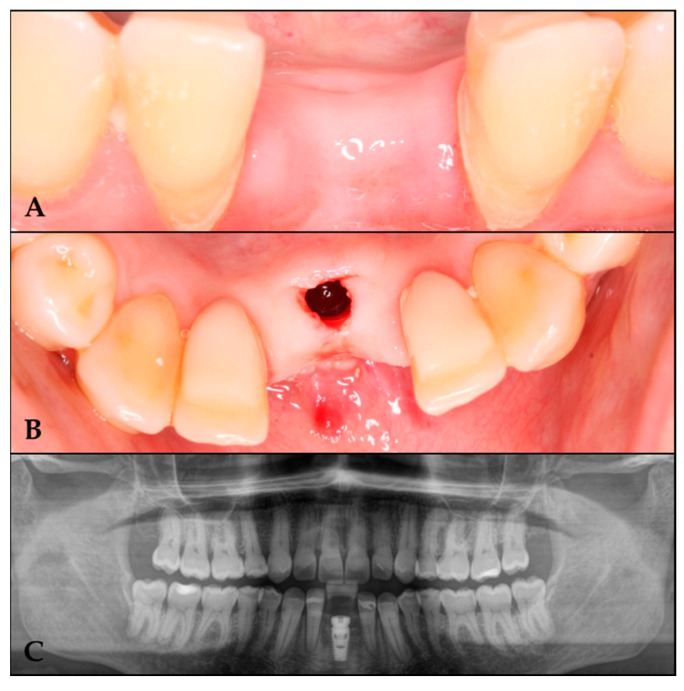
Implant placement in the regenerated area. (**A**) Regenerated area at 10 months. (**B**) Implant in the regenerated area. (**C**) Orthopantomography with the implant in the regenerated area.

Osteotomy preparation was performed according to the planned implant dimensions (Figure 5), followed by placement of a C1 MISS implant (3.75 mm diameter × 10 mm length) in the prosthetically driven position to fulfill restorative requirements.

### 3.3. Variables Analyzed

#### 3.3.1. Radiographic Measurements

CBCT scans of the entire arch were taken 21 days before surgery and 8 months afterward. CT data were exported in DICOM format. The DICOM data were analyzed using 3DSlicer medical software version 5.2.1 (https://slicer.org accessed on 9 February 2023) [17]. Preoperative and postoperative studies were segmented using the software’s thresholding tool, and the resulting segments were superimposed. The difference between the preoperative and postoperative segments was re-segmented to obtain the graft segment, which was then exported in STL format [18].

**Figure 6:**
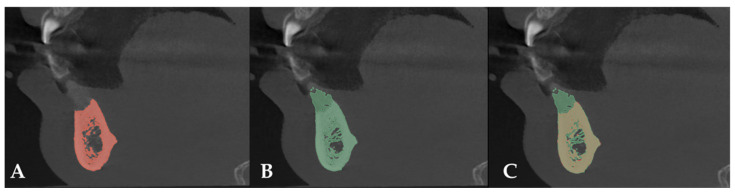
(**A**) Preoperative CT scan. (**B**) Postoperative CT scan. (**C**) Superimposition of preoperative and postoperative CT scans.

After comparing preoperative and postoperative CT images, an increase of 8.80 mm in height and 7.6 mm in bone width was observed. Bone regeneration was confirmed (Figure 6).

#### 3.3.2. Histological Analysis

The obtained sample will be placed in a 10% formalin solution. The tissues will be embedded in paraffin, and 5-micron sections will be prepared and stained with hematoxylin and eosin for histological analysis to determine the degree of bone formation.

− Macroscopy:

We receive a cylinder of 2 × 8 mm in the laboratory, fixed in formalin, with a firm density and a whitish colour.

The paraffin processing was made after a decalcification on formic acid. The sample was cut in a microtome MICROM HM 355S at 4 microns section. The slides were stained with the hematoxylin and eosin stain and the Gömöri trichrome stain and visualized with a microscope, NIKON Eclipse, provided with a camera and image analysis system.

− Microscopy:

The sample was representative of neoformed bone attached to gingival mucosa. The mucosal surface was covered with multiple layers of stratified squamous epithelial tissue, well organized and with maturation towards the surface, covered with a thin parakeratotic corneal layer. The underlying stroma has dense collagen, is well vascularized, and is infiltrated by some mature perivascular lymphocytes.

There is a periosteal transition towards the neoformed bone tissue, with some osteoblasts on the surface. The bone is of trabecular type, with thick trabeculae of mixed, laminar, and reticular type of bone, mineralized. There is also stroma with a rich vascularization. There is no cellular inflammation.

Assuming that the bone tissue has an isochoric distribution, we used point count girds of the stereology method of image analysis to calculate the relative volume occupied by the different components, with the result of a 31% of bone total density, 15% of inorganic residual material, a 40% of fibroadipous intertrabecular tissue, and a median density of 5 osteocytes/10,000 square microns. There are viable osteoblasts, with a mean of 6.2 per square millimeter.

**Figure 7:**
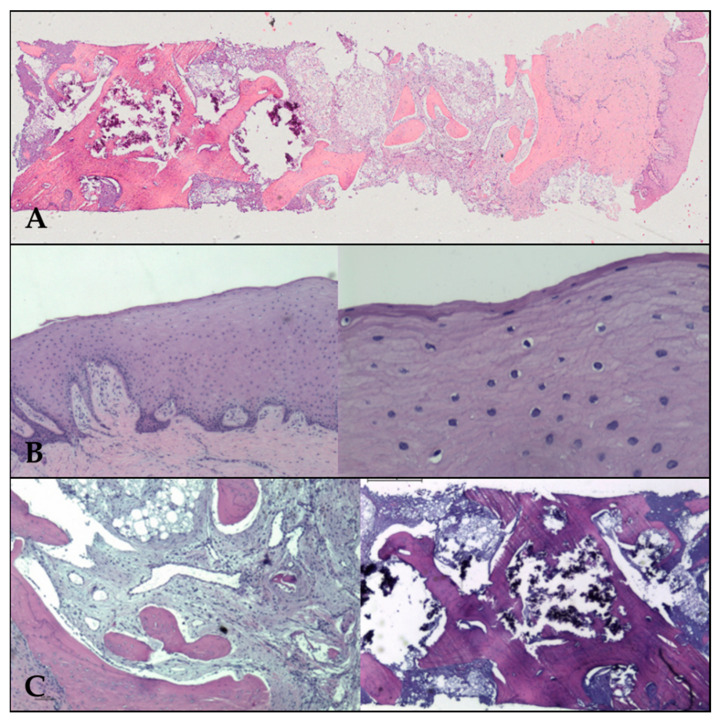
(**A**) Panoramic view of neoformed bone, covered by neoformed gum with epithelia on the Surface. (**B**) Epithelial mucosa at ×2 (left) and ×20 (right) low power fields. The corneal layer is evident on the Surface on the right. (**C**) Left: the stroma of fibroadipous tissue (×4 magn.) in the intertrabecular spaces. Right: inorganic material (×2 magn.).

The final diagnosis was that of “sample of neoformed bone, obtained from zone of 31, formed by mature spongious bone, mineralized, viable, with intertrabecular spaces occupied by well vascularized fibroadipous tissue and a residual component of basophilic amorphous inorganic material, calcified” (Figure 7).

## 4. Fixed Prosthesis Placement

**Figure 8:**
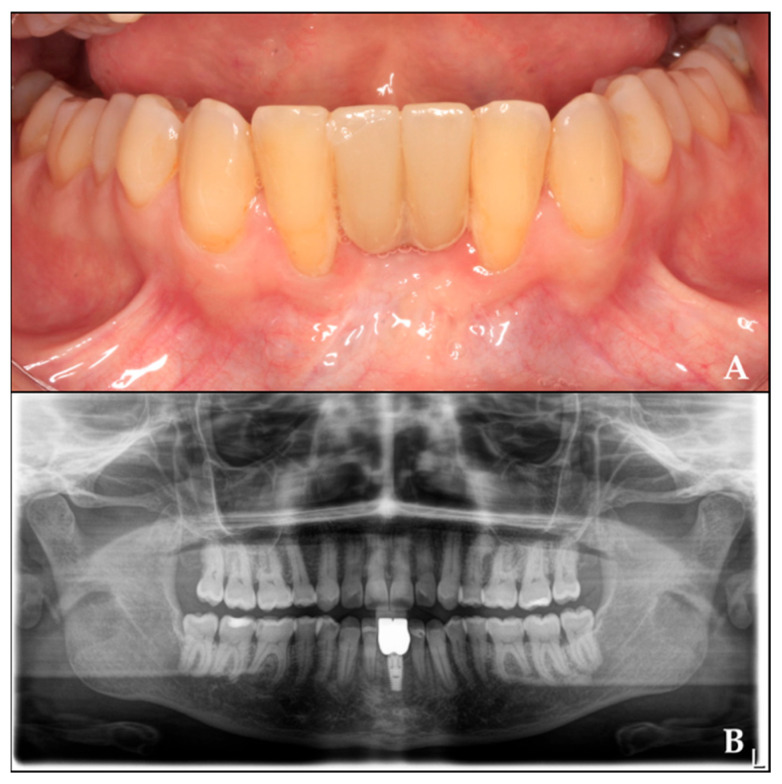
Rehabilitation of the regenerated area with crowns twenty months after implant placement. (**A**) Prosthetic rehabilitation in the regenerated area. (**B**) Orthopantomography with crowns over the implant.

Four months after implant placement, the prosthetic restoration was placed. After a two-year follow-up, the radiograph shows the final outcome (Figure 8).

## 5. Discussion

This clinical case demonstrates a successful approach to guided bone regeneration, achieving 8.8 mm of bone height gain and 7.6 mm of width, as well as the formation of keratinized gingiva.

We selected tricalcium phosphate as biomaterial of choice for serving as an optimal scaffold for blood clot stabilization, facilitating cellular migration and osteoprogenitor cell differentiation, which are critical for bone neoformation [15]. Its biphasic resorption kinetics (α-TCP: rapid; β-TCP: gradual) aligns with the bone remodeling timeline, ensuring progressive replacement by native bone without compromising structural integrity during the regeneration process [19].

Histomorphometric evaluation demonstrated successful osseous regeneration, evidenced by the presence of mature, mineralized bone suitable for implant placement and subsequent osseointegration. Furthermore, histological assessment identified well-organized gingival mucosa with stratified squamous epithelium, confirming the development of keratinized tissue. This finding holds particular clinical significance, as current consensus strongly advocates for adequate keratinized gingiva to maintain peri-implant health and long-term stability [20,21]. While conventional GBR procedures frequently compromise soft tissue architecture—necessitating additional surgical interventions to establish proper keratinized tissue—our technique achieved both hard and soft tissue regeneration simultaneously, potentially eliminating the need for secondary procedures.

The majority of GBR techniques, whether using membranes or block bone grafts, rely on the four PASS principles [22] to ensure surgical success and minimize complications. Managing both soft and hard tissues demands greater surgical expertise, making the procedure more technically demanding [23].

The innovative technique presented in this study challenges conventional PASS principles by eliminating the requirement for primary closure, as soft tissue coverage over the barrier proves unnecessary. This modification not only simplifies the surgical procedure but also permits continuous clinical monitoring of the guided bone regeneration process. Unlike traditional GBR approaches that frequently require autogenous bone harvesting—with its associated donor site morbidity and risk of graft exposure [24]—our technique achieves comparable regenerative outcomes through the combination of a patient-derived blood clot and tricalcium phosphate, avoiding secondary surgical sites. The barrier was intentionally placed near the tooth without resulting in any infectious complications or adverse effects. The non-submerged design allowed for direct visual monitoring of the regeneration site, reducing risks associated with flap closure.

While established methods such as Urban’s approach (using either resorbable membranes or PTFE) [25] and the Khoury technique [19] have demonstrated reliable bone gain outcomes, titanium occlusive barriers offer distinct clinical advantages. Our results show this approach provides similarly effective vertical (8.8 mm) and horizontal (7.6 mm) bone regeneration [13], while being significantly less invasive. The decision to employ any particular regenerative technique should be made after careful consideration of three factors: the specific bone defect characteristics, individual patient anatomy and biology, and the surgeon’s technical expertise with the procedure.

## 6. Conclusions

Further research is necessary to validate this technique, as conclusions cannot be generalized based on a single case. However, the results suggest that this approach may represent a significant advancement in GBR, as it enables both bone regeneration and keratinized gingiva formation in a single surgery. Additionally, it simplifies the procedure for the operator and reduces morbidity for the patient.

The use of a digitally designed occlusive barrier allowed for better adaptation to the bone defect, making the placement process easier for the surgeon while also reducing morbidity by eliminating the need for a donor site. This approach facilitates effective guided bone regeneration while improving overall clinical outcomes.

## Data Availability

The data supporting this study are openly available in the Redcap data repository at the Biomedical Research Institute of Malaga (IBIMA).

## Abbreviations

CAD	Computer-Aided Design
CBCT	Cone Beam Computed Tomography
GBR	Guided Bone Regeneration
STL	Stereolithography
SSS	Sterile Saline Solution
DICOM	Digital Imaging and Communication in Medicine
PTFE	Polytetrafluoroethylene
